# Intensive Care and Treatment of Severe Guillain–Barré Syndrome

**DOI:** 10.3389/fphar.2021.608130

**Published:** 2021-04-27

**Authors:** Pei Shang, Jiachun Feng, Wei Wu, Hong-Liang Zhang

**Affiliations:** ^1^ Department of Neurology, First Hospital of Jilin University, Changchun, China; ^2^ Department of Molecular Pharmacology and Experimental Therapeutics, Mayo Clinic College of Medicine and Science, Rochester, MN, United States; ^3^ Department of Neurosurgery, First Hospital of Jilin University, Changchun, China; ^4^ Department of Life Sciences, National Natural Science Foundation of China, Beijing, China

**Keywords:** Guillain–Barré syndrome, intensive care, intravenous immunoglobulin, mechanical ventilation, plasma exchange

## Abstract

Guillain–Barré syndrome (GBS) is an acute polyneuropathy mostly characterized by acute flaccid paralysis with or without sensory/autonomous nerve dysfunction. Current immuno therapies including intravenous immunoglobulin (IVIg), plasma exchange (PE), and newly developed biological drugs benefit patients by alleviating hyperreactive immune responses. Up to 30% of patients develop respiratory failure during hospitalization and require mechanical ventilation and intensive care. Immunotherapies, mechanical ventilation, supportive care, and complication management during the intensive care unit (ICU) stay are equally emphasized. The most important aspect of intensive care and treatment of severe GBS, that is, mechanical ventilation, has been extensively reviewed elsewhere. In contrast to immunotherapies, care and treatment of GBS in the ICU setting are largely empirical. In this review, we intend to stress the importance of intensive care and treatment, other than mechanical ventilation in patients with severe GBS. We summarize the up-to-date knowledge of pharmacological therapies and ICU management of patients with severe GBS. We aim to answer some key clinical questions related to the management of severe GBS patients including but not limited to: Is IVIg better than PE or *vice versa*? Whether combinations of immune therapies benefit more? How about the emerging therapies promising for GBS? When to perform tracheal intubation or tracheostomy? How to provide multidisciplinary supportive care for severe cases? How to avert life-threatening complications in severe cases?

## Introduction

Guillain–Barré syndrome (GBS) is recognized as a paralytic peripheral neuropathy with an annual incidence of 0.81–1.89 cases (median, 1.11) per 100,000 persons worldwide (Benedetti et al., 2019). The in-hospital mortality rate of GBS is approximately 2.6–2.8%, and risk factors include severity of weakness at entry, time to peak disability, mechanical ventilation (MV), old age, and pulmonary and cardiac complications (Alshekhlee et al., 2009; Van Den Berg et al., 2013). Most patients with GBS are clinically characterized by acute flaccid paralysis and/or sensory/autonomous nerve dysfunction (Sejvar et al., 2011). Almost two-thirds of GBS cases have a prodromal upper respiratory tract or gastrointestinal tract infection (Wakerley and Yuki, 2013). Prognostic factors of a poor prognosis mainly include old age, acute hospital stay, prolonged MV and intensive care unit (ICU) stay, and insufficient rehabilitation after discharge (Khan et al., 2010; Khan and Amatya, 2012; Van Den Berg et al., 2018). Patients’ recovery benefits from high-intensity multidisciplinary ambulatory rehabilitation even up to 12 months since onset, highlighting the importance of early and persistent rehabilitation (Khan and Amatya, 2012).

**FIGURE 1:**
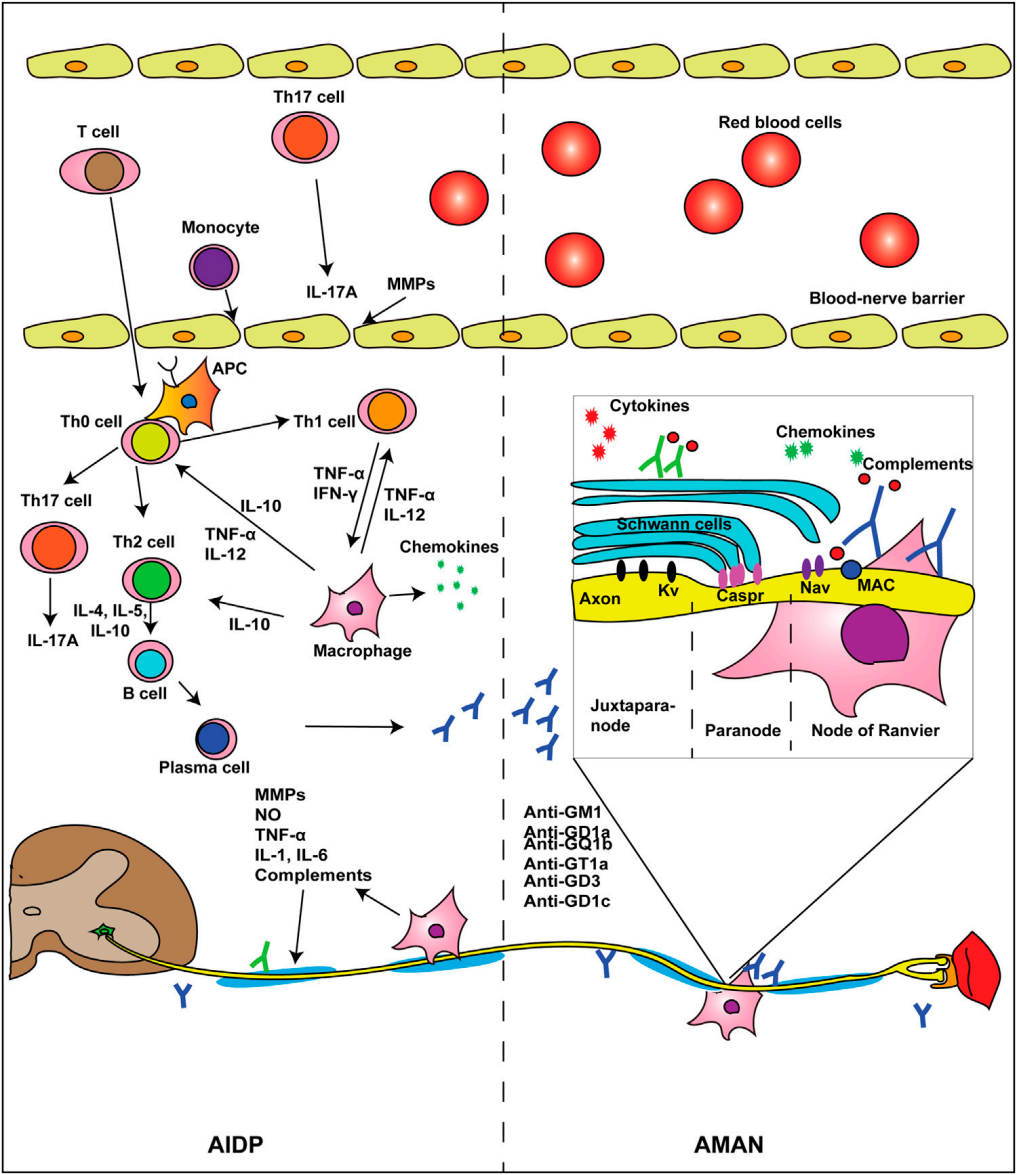
Pathogenesis of AIDP and AMAN. The pathogenesis varies among different subtypes of GBS. In AIDP, Th0 cells go across the BNB and develop into Th1, Th2, or Th17 cells after stimulated by APCs. Th1 cells release TNF-α and IFN-γ, facilitating macrophage recognizing Schwann cells and stripping off the myelin sheath. Th2 cells promote the proliferation and differentiation of B cells. Mature B cells develop into plasma cells and produce pathogenic antibodies. Activated macrophages interact with immune cells and cause a storm of inflammation-associated chemokines and cytokines. In AMAN, antiganglioside antibodies recognize the node of Ranvier and cause axonal degeneration via MAC. Some autoantibodies may also target Schwann cells and trigger demyelination. Abbreviations: AIDP, acute inflammatory demyelinating polyneuropathy; AMAN, acute motor axonal neuropathy; APC, antigen-presenting cell; BNB, blood–nerve barrier; Caspr, contactin-associated protein; FasL, Fas–Fas ligand; GBS, Guillain–Barré syndrome; IFN-γ, interferon γ; IL, interleukin; Kv, voltage-gated potassium channels; MAC, membrane attack complex; MMPs, metalloproteinases; Nav, voltage-gated sodium channels; NO, nitrogen oxide; TGF-β, transform growth factor-β; Th cell, T helper cell; TNF-α, tumor necrosis factor-α.

GBS pathologically affects the peripheral nervous system (PNS) and is classified into several subtypes according to the distinct clinical and pathological features (Table 1). Acute inflammatory demyelinating polyneuropathy (AIDP) due to acute inflammatory responses and demyelination of the peripheral nerves is prototypical of GBS (Hughes, 2020). Axonal variants mainly including acute motor axonal neuropathy (AMAN) and acute motor sensory axonal neuropathy (AMSAN) predominate in Asian countries (Feasby et al., 1986; Peric et al., 2014; Benedetti et al., 2019). Molecular mimicry between *Campylobacter jejuni* lipo-oligosaccharide and host gangliosides elicits hyperreactive immune responses and cytokine storm, which has been accepted to explain the pathogenesis of *Campylobacter jejuni*–associated GBS (Soltani et al., 2019). Other pathogens and noninfectious triggers like surgery, trauma, and intravenous use of gangliosides have also been reported (Shang et al., 2020a). The pathogenesis of AIDP and AMAN is briefly depicted in Figure 1. As pathological studies of axonal GBS reveal deficits in both axons and excitable axolemma, a new definition, namely, nodo-paranodopathy represents an updated understanding of the axonal variants of GBS (Uncini et al., 2013). Regional variants of GBS like Miller Fisher syndrome (MFS), with a triad of ophthalmoplegia, ataxia, and areflexia (without limb weakness), usually manifest as a self-limited clinical course (Overell et al., 2007).

**TABLE 1:** Differentiation between AIDP, AMAN, and AMSAN.

GBS subtype	AIDP	AMAN	AMSAN
Clinical features	Progressive para-/tetraparesis; sensory deficits; hypo- or areflexia, with or without cranial nerve; over-month recovery	Mainly motor deficiency; uncommonly with cranial nerve symptoms (<20%); without pain or sensory loss; with absent tendon reflex (normal or even exaggerated reflexes may exist in the early phase or atypical cases); with rapid or slow recovery	Similar to AMAN but with sensory deficits; usually with a severe disease course
Electrophysiological results	Slowed sensorimotor nerve conductions or CBs, excessive temporal dispersions of CMAPs, and a prolonged DML or F-wave latency	Usually no evidence for AIDP (may show segmental conduction blocks in an early phase); show RCFs or decreased CMAP amplitudes	Axonal polyneuropathy features with sensory attenuated or absent action potential
Antibody classification	Not routinely detectable	Mainly GM1 and GD1a	Same as AMAN
Involved nerves	Sensorimotor, cranial, and/or autonomic nerves	Motor nerves	Motor and sensory nerves

Abbreviations: AIDP, acute inflammatory demyelinating polyneuropathy; AMAN, acute motor axonal neuropathy; CB, conduction block; CMAP, compound muscle action potential; DML, distal motor latency; RCF, reversible conduction failure.

Hypoactive or absent deep tendon reflexes are a common clinical feature of GBS, although increased or normal tendon reflexes can be seen in about 10% of patients during the early phase of the disease (Yuki, 2012; Leonhard et al., 2019). Albuminocytologic dissociation in the cerebrospinal fluid (CSF) is a clinical hallmark of GBS, which appears in up to 90% of all patients during the third week of the disease course (Dimachkie and Barohn, 2013). An electrophysiological study performed 3–4 weeks after onset can differentiate axonal GBS from AIDP (Rajabally et al., 2015). The efficacy of intravenous immunoglobulin (IVIg) and plasma exchange (PE) in the treatment of GBS has been validated by extensive investigations (Hughes et al., 2014). Notwithstanding, these immunotherapies for GBS are still incurring controversies due to its high cost, potential adverse events, and incompletely known mechanisms, among others (Hughes et al., 2007).

Up to 30% of GBS patients require MV as well as ICU admission (Yuki and Hartung, 2012; Van Den Berg et al., 2018). Whenever feasible, rapidly progressing patients who are unable to walk without aid, are bedbound, or have labile blood pressure, cardiac arrhythmia, or respiratory distress should be treated immediately, preferably in an ICU (Harms, 2011; Verboon et al., 2017). Failure to refer severely affected patients to a specialized neurological ICU may lead to higher mortality rates, implicating the importance of earlier referral of severe cases and providing neurocritical care (Taylor et al., 2017). A multidisciplinary team is encouraged to provide supportive care for severe GBS cases in the ICU to avoid multiple comorbidities.

**FIGURE 2:**
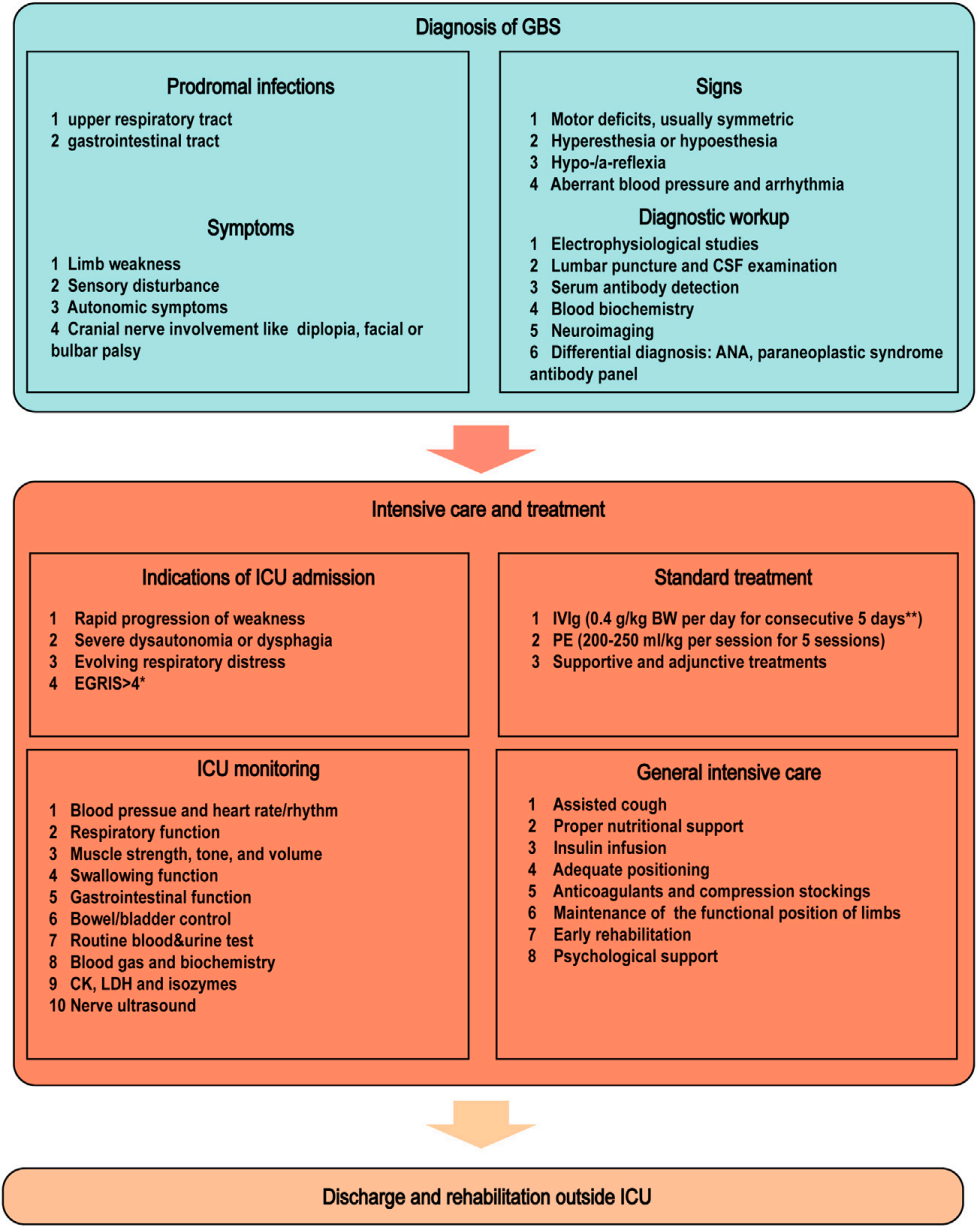
ICU admission and management of severe GBS. The diagnosis of GBS is based on the prodromal infections, symptoms, and signs combined with laboratory workups including electrophysiological studies and CSF/serum tests. Pharmacological treatments should be applied immediately in progressive GBS patients who cannot walk without aid. Decision-making as to ICU admission should be based on EGRIS, respiratory function, and extent of dysautonomia/dysphagia. The ICU monitoring of physiological parameters facilitates the recognition of GBS progression and the decision-making process for neurointensivists. *EGRIS is calculated *via* integrating MRC scores, facial/bulbar weakness, and duration from the onset to admission. **IVIg at 2g/kg BW can even be completed within 2 or 1 day in heathy cohorts, especially for young patients with normal cardiac and renal functions. Abbreviations: ANA, antinuclear antibody; BW, body weight; CK, creatine kinase; CSF, cerebrospinal fluid; EGRIS, Erasmus GBS respiratory insufficiency score; GBS, Guillain–Barré syndrome; ICU, intensive care unit; IVIg, intravenous immunoglobulin; LDH, lactate dehydrogenase; MRC, Medical Research Council; PE, plasma exchange.

In this narrative review, we will summarize the updated knowledge on the intensive care and treatment of patients with severe GBS, with a focus on canonical therapies and promising areas in pharmacological interventions, MV-associated decision-making, and ICU care (Figure 2). As the incidence of GBS is low and the disease is clinically heterogeneous, most of the documented investigations have a relatively small sample size. In contrast with immunotherapies, care and treatment of GBS in the ICU setting are largely empirical. As a consequence, observational data are occasionally used to guide clinical practice.

## Canonical and Emerging Immunotherapies of Guillain–Barré Syndrome

**FIGURE 3:**
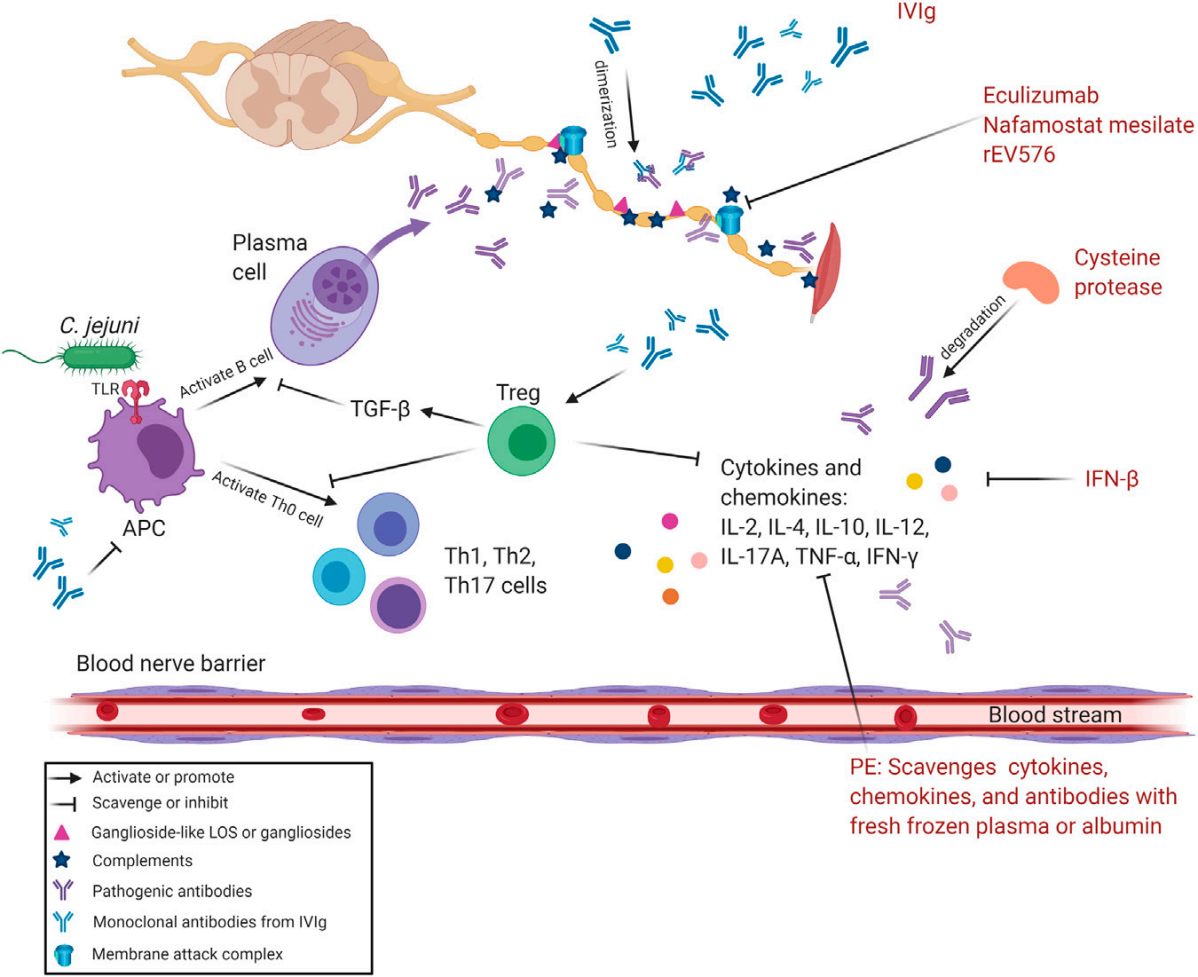
Pharmacological therapeutic targets of GBS. The hyperreactive cellular and/or humoral immune responses in GBS are the main targets of current pharmacological interventions. IVIg can inhibit the production of pathogenic antibodies and pro-inflammatory mediators released by T helper cells and activated B cells *via* functioning on Tregs. IVIg also promotes the dimerization of antiganglioside antibodies and inhibits APCs to alleviate immune responses. PE mainly replaces plasm rich in antiganglioside antibodies and pro-inflammatory mediators with fresh frozen plasma/albumin. Eculizumab, nafamostat mesilate, and rEV576 are complement inhibitors that can prevent MAC formation. IFN-β attenuates inflammation induced by cytokines and chemokines. Cysteine protease degrades pathogenic antibodies and mitigates hyperreactive immune responses. Abbreviations: APC, antigen-presenting cell; C. jejuni, *Campylobacter jejuni*; GBS, Guillain–Barré syndrome; IFN-β, interferon β; IL, interleukin; IVIg, intravenous immunoglobulin; LOS, lipo-oligosaccharide; MAC, membrane attack complex; PE, plasma exchange; TGF-β, transform growth factor β; Th cell, T helper cell; TLR, Toll-like receptor; TNF-α, tumor necrosis factor-α; Treg, regulatory T cell.

Immunotherapies were originally postulated from the immune-related pathogenesis in GBS: IVIg dimerizes pathogenic autoimmune antibodies (Verboon et al., 2017); PE scavenges pathogenic inflammatory mediators (Chevret et al., 2017); corticosteroids suppress hyperreactive autoimmunity (Wang et al., 2015b). IVIg and PE have been the mainstay for the treatment of GBS (Chevret et al., 2017) (Table 2). Currently, IVIg and PE are used to treat up to 92% of GBS patients in the United States (Verboon et al., 2019). However, little evidence supports their use in patients with mild GBS, treatment failure, and treatment-related fluctuation (TRF) (Verboon et al., 2019). We illustrate potential pharmaceutical targets based on the pathogenesis of GBS in Figure 3.

**TABLE 2:** Comparisons between IVIg and PE.

	IVIg	PE
Regimen	0.4 g/kg BW per day for 5 consecutive days^a^	40–50 ml plasma/kg BW per session for 5 sessions in 7–14 days
Mechanism	Inhibits Fc-mediated macrophage activation, prevents binding of antibodies to neural targets, prevents complement activation, and dimerizes antiganglioside IgG antibodies (Verboon et al., 2017)	Removes hyperreactive immune-associated antiganglioside antibodies and pro-inflammatory cytokines with albumin or fresh frozen plasma (Chevret et al., 2017)
Efficacy	Diminishes pathogenic antibodies, machine-independent and easy delivery, and effective especially in pediatric cases (Hughes et al., 2014)	Removes pathogenic antibodies without frozen plasma; hastens recovery; shortens MV and hospitalization; and effective in treating AMAN (Chevret et al., 2017)
Advantages (Hughes et al., 2014; Greene-Chandos and Torbey, 2018)	Prevents nosocomial pneumonia and infections, provides more comfort, easy to initiate, and convenient to infuse *via* the peripheral veins	Substitutes IVIg in patients refractory to IVIg treatment or with IVIg contraindications (i.e., allergic to IVIg and selective IgA deficiency)
Disadvantages (Greene-Chandos and Torbey, 2018)	May need a second dose of IVIg for TRF, no long-term benefits, and contraindicated in patients with renal deficiency or congestive heart failure	Relatively more expensive, might dilute antiinfectious immunoglobulins when only albumin is used, needs an experienced team, TRF,^b^ marked dysautonomia, and contraindicated in patients with septic shock or myocardial infarction within 6 months
Complications (Koichihara et al., 2008; de Havenon et al., 2014; Nguyen et al., 2014; Stetefeld et al., 2014; Greene-Chandos and Torbey, 2018; Baudel et al., 2020)	Stroke, PRES, aseptic meningitis, venous embolism, allergic reaction, splenic rupture, and hemolytic anemia; infusion-related complications including TRALI^c^, fatigue, fever, and nausea	Central venous access complications, pneumonia, hypocalcemia-associated paresthesia, transfusion reactions, abnormal clotting and DVT, hypotension, allergic reaction, pneumothorax, and hemolysis
Hospitalization cost (Beydoun et al., 2020)	$103,223	$149,143
Hospital stay (Beydoun et al., 2020)	10.24 days	17.78 days

Abbreviations: AMAN, acute motor axonal neuropathy; BW, body weight; DVT, deep vein thrombosis; GBS, Guillain–Barré syndrome; IVIg, intravenous immunoglobulin; MV, mechanical ventilation; PE, plasma exchange; PRES, posterior reversible encephalopathy syndrome; TRALI, transfusion-related acute lung injury; TRF, treatment-related fluctuation.

^a^ Or 1 g/kg BW for 2 days or 2 g/kg BW for 1 day

^b^ TRF refers to improvement in the Hughes functional grading scale (HFGS) score of at least one grade after completion of immunotherapy followed by worsening of the HFGS score of at least one grade within the first 2 months after disease onset (Kleyweg and Van Der Meche, 1991).

^c^ TRALI is a rare and devastating complication of transfusion, which is defined as acute-onset respiratory distress after administration of blood products (Baudel et al., 2020). Presumably, IVIg-associated TRALI may implicate accelerated deterioration or worse outcomes in a subgroup of IVIg-treated GBS patients.

### Pharmacological Mechanism, Therapeutic Regimen, and Side Effects of IVIg

IVIg is a plasma product that contains a broad spectrum of different antibodies. IVIg has pleiotropic immunomodulatory effects, which include inhibiting Fc-mediated activation of macrophages, preventing the binding of antibodies to neural targets, and preventing complement activation which would otherwise trigger further nerve damage (Verboon et al., 2017). Dimerization of antiganglioside IgG antibodies induced by IVIg alleviates their immunoreactivity in GBS patients’ sera (Svacina et al., 2019). Meanwhile, high-dose IVIg [ranging from 1000 to 3000 mg/kg body weight (BW)] results in immunosuppressive and anti-inflammatory phenotypes, which is universally employed to treat autoimmune diseases like GBS (Arumugham and Rayi, 2020).

IVIg is most often prescribed in GBS due to its simple procedure and machine-independent attribute (Beydoun et al., 2020). However, IVIg is contraindicated in patients who are hypersensitive to the active substance in the product or have a previous history of severe systemic or anaphylactic responses to IVIg or have anti-IgA antibodies and selective IgA deficiencies. Unless contraindicated, patients unable to walk without assistance are routinely treated with a standard IVIg regimen (0.4 g/kg BW per day, for five consecutive days, or 1 g/kg BW per day, for two consecutive days). Not only is IVIg easier to administer but it also efficiently hastens recovery (Hughes et al., 2014). Although a 2 g/kg BW IVIg course can be completed as fast as within a single day, fast infusion of immunoglobulin may increase colloidal osmotic pressure and possibly trigger cardiac arrest or renal failure (Tansley and Hall, 2015). Some patients may nonetheless continue to deteriorate or experience fluctuations of symptoms after the initial dose, necessitating the consideration of a second course of IVIg administration (Hughes et al., 2007; Verboon et al., 2017). The benefit of a second course of IVIg has yet to be corroborated (Walgaard et al., 2018; Verboon et al., 2020), although a minor increase in the serum IgG level was proposed as a predictor for poor outcomes after a single dose of IVIg (Kuitwaard et al., 2009). IVIg is correlated with adverse events including stroke, hemolytic anemia, transfusion-related acute lung injury (TRALI), aseptic meningitis, and venous embolism (Koichihara et al., 2008; Nguyen et al., 2014; Stetefeld et al., 2014; Baudel et al., 2020) (Table 2).

### Mechanism of Action, Adverse Effects, and Optimization of PE

PE was the first proven immunotherapy for GBS, followed by IVIg. Currently, PE is used as an effective therapy to promote recovery of GBS patients (El-Bayoumi et al., 2011). PE was administered in around 4% GBS patients worldwide, except in several countries (i.e., the United States. 15%, Malaysia 33%, and Italy 30%) (Verboon et al., 2019). Of the IVIg-treated patients without clinical improvements, 35% received a second immunotherapy and one-third in this cohort shifted to PE (Verboon et al., 2019). In practice, PE is strongly recommended for GBS patients in the acute phase with impaired independent walking capacity or requiring MV assistance, whereas contraindicated in patients who cannot tolerate central line placement or with unstable hemodynamics or allergic to frozen plasma/albumin.

PE mainly functions *via* scavenging pathogens and autoimmune antibodies in patients’ peripheral blood (Chevret et al., 2017). Patients with GBS routinely benefit from a standard PE schedule (5 sessions with 40–50 ml plasma/kg per session within 7–14 days) (Chevret et al., 2017). Usually, PE is performed every other day to allow the redistribution of pathogenic agents in both extravascular and intravascular compartments (Fernandez-Zarzoso et al., 2019). Efficacy of PE is closely dependent on the speed of production and clearance of pathogenic agents; as such, immunosuppressive treatments are regularly considered as adjuvants for PE (Fernandez-Zarzoso et al., 2019). Moderate-quality evidence shows higher efficacy of PE than supportive care alone in adults with GBS, without an significant increase in serious adverse events (Chevret et al., 2017). Nonetheless, adverse events of PE are occasionally reported including catheter-related infection, deep venous thrombosis (DVT), hypotension, septicemia, anaphylaxis, and hemolysis (Gwathmey et al., 2014). Common complications include headache, chills, myalgias, noncardiac chest pain, and aseptic meningitis (Gwathmey et al., 2012). In this regard, double filtration plasmapheresis appears safer and more efficient in removing specific antibodies and does not require fresh frozen plasma (Lin et al., 2015). Noticeably, although hypotension, coagulation disorders, or allergic reactions may occasionally occur, most adverse effects are unpredictable and PE is generally safe for patients in the ICU setting (Lemaire et al., 2017).

Optimization of the procedure of PE is intriguing. For instance, the appropriate frequency of PE is set as four sessions for moderate to severe GBS cases, while two sessions for those with mild GBS (French Cooperative Group on Plasma Exchange in Guillain-Barre Syndrome, 1997). PE can also be conducted with albumin and gelatin, instead of fresh frozen plasma (French Cooperative Group on Plasma Exchange in Guillain-Barre syndrome, 1987). However, when albumin or gelatin is used to replace patients' serum, dilution of the antiinfectious immunoglobulins needs caution (Shumak and Rock, 1984; Chevret et al., 2017). Between individual sessions, PE is suggested to be performed with continuous flow machines, instead of the intermittent version. However, the benefits of continuous flow machines in PE remain controversial (Mckhann, 1985; French Cooperative Group on Plasma Exchange in Guillain-Barre Syndrome, 1987). Taken together, PE is efficient in removing pathogenic agents from patients’ peripheral blood, whereas the procedure is complicated and machine-dependent.

### Comparisons Between IVIg and PE

Since the proof of concept for IVIg historically followed that of PE, the efficacy of IVIg has been compared to PE, rather than to placebo in randomized controlled trials (RCTs). IVIg (0.4 g/kg BW daily for five consecutive days) and PE (200–250 ml plasma/kg BW in five sessions) are equally effective for GBS (Hughes et al., 2014). In severe cases, IVIg started within 2 weeks from disease onset hastens recovery as much as PE (Hughes et al., 2014). IVIg and PE appear to carry comparable risks of adverse events, although early studies showed that PE was more likely than IVIg to be discontinued. The relatively complicated procedure of PE can be better completed by a specialized team (El-Bayoumi et al., 2011; Hughes et al., 2014).

Interestingly, mechanically ventilated adult GBS patients with IVIg infusion exhibit shorter hospitalization and earlier weaning and motility recuperation compared with those receiving PE, suggesting the superiority of IVIg to PE in the ICU (Charra et al., 2014). Conversely, PE appears superior to IVIg in MV-dependent pediatric GBS cases in light of the duration of MV (El-Bayoumi et al., 2011). In the United States, PE is associated with longer hospitalization (17.78 vs. 10.24 days), increased in-hospital mortality (3.8 vs. 1.4%), and greater hospitalization cost ($149,143 vs. $103,223) as compared with IVIg (Plasma Exchange/Sandoglobulin Guillain-Barre Syndrome Trial Group, 1997; Beydoun et al., 2020). In Bangladesh, a full course of IVIg costs about $ 12,000–16,000 for a 60-kg adult, whereas conventional PE within 5 days costs about $ 4,500–5,000 (Islam et al., 2018). In China, the cost for a full course of IVIg is $ 3,771 for a 60-kg adult.

In practice, patients with mild forms of GBS, TRFs, or treatment failures are frequently treated, despite the absence of evidence (Verboon et al., 2019). In particular, around 68% of patients with TRFs are re-treated with IVIg/PE, although inconsistent conclusions have been drawn from clinical observations (Van Doorn et al., 2010; Walgaard et al., 2018). Of note, the pharmacological impacts of IVIg/PE on renal function should be monitored especially in the critically ill population (Tansley and Hall, 2015). RCTs are needed to address clinical dilemmas, especially in choosing appropriate immunotherapies for patients with diverse GBS subtypes and circumstances. Theoretically, the use of PE followed by IVIg can be safer and more effective in treating patients with GBS. Consistently, administration of IVIg combined with PE could reduce GBS mortality, shorten hospitalization, and promote earlier weaning from MV in pediatric cases (Kesici et al., 2019). Nonetheless, the combination of IVIg and PE confers an insignificant advantage in adults (Plasma Exchange/Sandoglobulin Guillain-Barre Syndrome Trial Group, 1997; Hughes et al., 2014).

### Are Steroids Useful as an Adjuvant Therapy or a Monotherapy?

Corticosteroids were recommended in treating patients with refractory or severe GBS as immunosuppressants (Shahar, 2006). The add-on use of corticosteroids had been expected to exert a surplus effect to augment IVIg benefits (Visser, 1994; Van Koningsveld et al., 2004). However, later observations suggest that corticosteroids given alone do not significantly hasten recovery or affect the long-term outcomes; according to some, oral corticosteroids delay recovery (Hughes et al., 2016). Compared with IVIg monotherapy, the add-on use of corticosteroids worsened the short-term prognosis of severely paralytic and MV-dependent GBS patients (Wu et al., 2015b). The detrimental effects on those patients from the add-on therapy might be attributed to hyperglycemia induced by corticosteroids (Hughes, 2004; Wu et al., 2015b). Corticosteroids may also dampen regeneration by reducing the scavenger functions of macrophages (Wu et al., 2015b). As such, corticosteroids do not remarkably hasten recovery from severe GBS or improve the long-term outcomes (Hughes et al., 2016). Collectively, corticosteroids are not recommended in for the general management of GBS. However, its efficacy in rare subtypes of GBS awaits further investigation.

### Adjuvant and Emerging Therapies

Currently, researchers are scrutinizing potential therapeutic targets and testing other immune therapies (i.e., anti–B-cell therapy, anticomplement therapy, and anticytokine therapy) on animal models or humans (Ostronoff et al., 2008; Misawa et al., 2018; Soltani et al., 2019). Newly developed biological medications like eculizumab have displayed considerable potency (Misawa et al., 2018). Besides, initiation, severity, and progression of GBS also correlate with nutritional factor abnormalities including serum folate and vitamin deficiency (Staff and Windebank, 2014; Gao et al., 2018). Acute axonal neuropathy could be triggered by alcoholism- or bariatric surgery-induced nutritional loss (Hamel and Logigian, 2018). Therefore, neurotrophic therapies, including vitamin supplementation, might benefit GBS outcomes. Notwithstanding, limitations and controversies are notable in the immunotherapies or nutrition supplements. Evidence is still lacking to support the efficacy of IFN-β1a, brain-derived neurotrophic factor (BDNF), CSF filtration with PE, *tripterygium* polyglycoside, and eculizumab in treating GBS due to low or very low certainty of evidence for the interventions and outcomes (Pritchard et al., 2016; Doets et al., 2020). Moreover, glycosylated ganglioside species including GM1, GD1a, GD1b, and GT1b predominate in the adult brain, which is contradictory to the classical immune hypothesis that regards antigens as unmodified proteins leading to technical challenges in the design of commercialized antibody-detecting kits (Shang et al., 2020a; Cutillo et al., 2020).

### MECHANICAL VENTILATION: A COMPLICATED DECISION-MAKING PROCESS

Respiratory muscle weakness (i.e., oropharyngeal, laryngeal, tongue, retropharyngeal, intercostal, and diaphragmatic weakness) in GBS patients contributes to the loss of airway protection, ineffective cough, and multiple pulmonary complications (Hahn, 2001). Meanwhile, bulbar palsy and dysautonomia deteriorate the secretion clearing process and further increase the risk of pulmonary infection and respiratory failure (Chakraborty et al., 2020). Importantly, compared with AIDP, patients with AMAN appear more likely to develop respiratory failure, although the difference is not significant (47.4%, 9/19 vs. 33.8%, 22/65) (Kalita et al., 2016).

**FIGURE 4:**
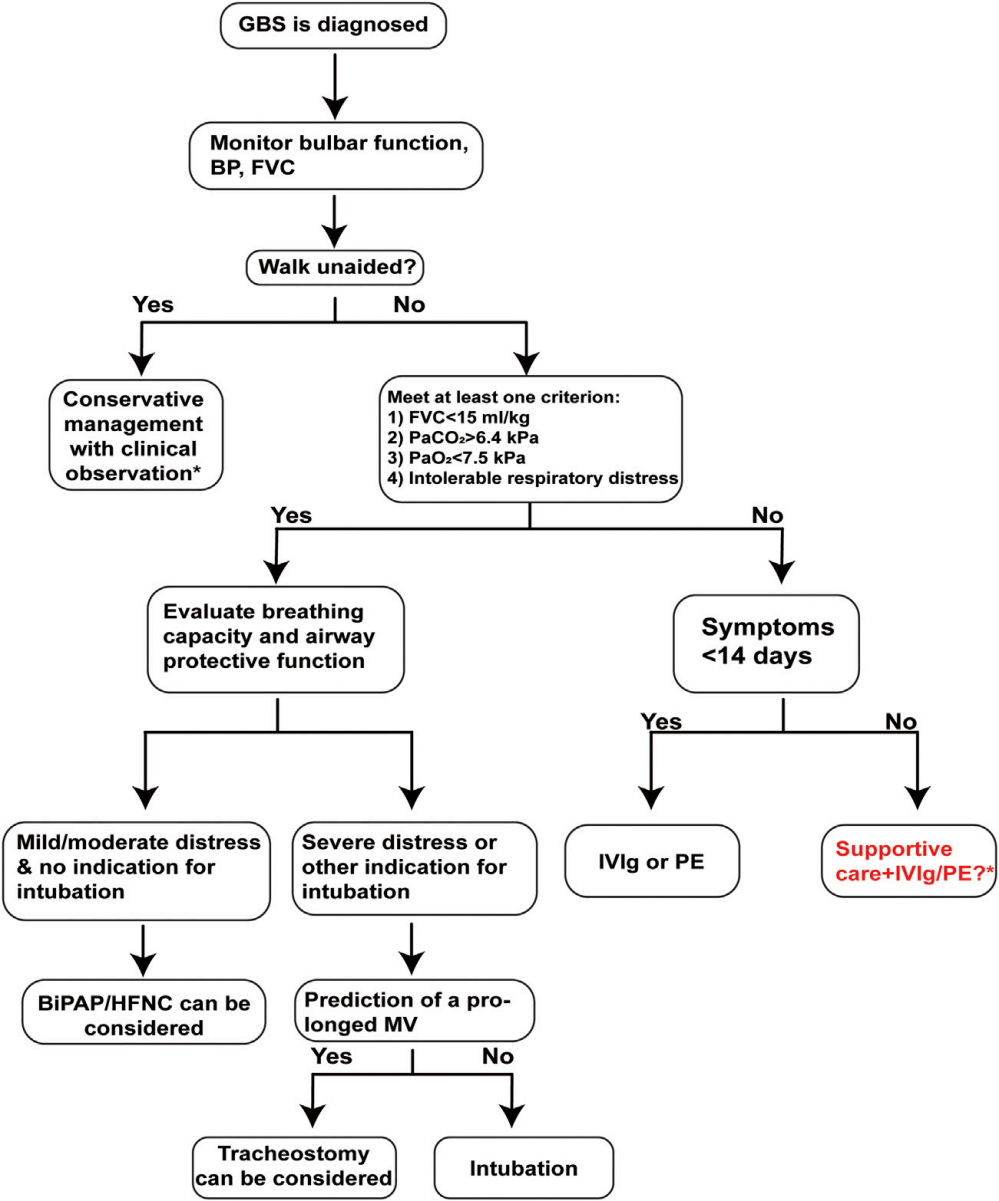
A pragmatic decision-making workflow for the intensive care and treatment of GBS. Clinical decision on MV is closely correlated to the prognosis of GBS patients. The strict screening steps rely on dynamic measurements of disease progression and respiratory functions, which are vital for the implementation of proper individualistic therapeutic strategies. A decision for noninvasive ventilation, tracheal intubation with MV, or tracheostomy relies on the respiratory capacity and airway protection capacity of GBS patients. *Treatment dilemmas occur in patients with relatively mild symptoms or when the plateau of weakness was more than 2 weeks before. Abbreviations: BiPAP, bilevel positive airway pressure; BP, blood pressure; EGRIS, Erasmus GBS respiratory insufficiency score; FVC, forced vital capacity; GBS, Guillain–Barré syndrome; HFNC, high-flow nasal cannula; ICU, intensive care unit; IVIg, intravenous immunoglobulin; MV, mechanical ventilation; PE, plasma exchange.

A multispecialty team is needed for the decision-making of MV in patients with respiratory failure. The key issues implicated in the initiation of MV, the configuration parameters of ventilators, and the decision on tracheostomy, etc*.* have been extensively reviewed elsewhere (Shang et al., 2020b). In practice, MV needs to be considered when one or two of following criteria are met: (a) vital capacity <15 ml/kg BW, (b) hypoxemia (PaO_2_ < 7.5 kPa), (c) hypercarbia (PaCO_2_ > 6.4 kPa), and (d) intolerable respiratory distress (Burakgazi and Hoke, 2010) (Figure 4). Notably, severe GBS cases with bulbar dysfunction and a VC of less than 20 ml/kg BW may indicate a rapid decline of neuromuscular function and a need for earlier ventilation (Lawn et al., 2001). Multivariate analyses on adult GBS patients not ventilated at admission reflected several candidate predictors for early ventilation including time from onset to admission of <7 days, inability to cough or stand, inability to lift the head or elbows, and increased liver enzymes (Sharshar et al., 2003). Indeed, earlier nadir and more rapid progression were observed in patients with AMAN than those with AIDP, suggesting that patients with AMAN are more likely to develop prolonged paralysis and respiratory failure over a few days (Hiraga et al., 2003). Early prediction of MV (Wu et al., 2015a) enables caregivers to tailor supportive care and individualized treatment so as to avoid complications including pneumonia and sepsis and to improve quality of life. However, in a recent RCT, GBS patients receiving early MV did not exhibit significant differences in the incidence of pneumonia, length of hospital stay, neurological scores, tracheostomy rate, and mortality as compared with those treated only with physiotherapy (Melone et al., 2020). Further investigations on the risk benefic ratio of early MV are warranted, in particular for patients with different subtypes of GBS.

Individualized monitoring of VC, respiratory rate and pressure, and oxygen saturation is emphasized in patients with severe GBS, especially for those with rapidly progressive paralysis (Chevrolet and Deleamont, 1991; Shang et al., 2020b). In all possibilities, MV should be initiated stepwise and individually based on patients’ oxygen saturation and respiratory efforts. For example, caregivers can initiate MV first at night only and then in the morning. Nocturnal MV has been reported to relieve chronic hypoventilation during disease progression and prolong survival (Annane et al., 2014). Meanwhile, 18- to 20-h prone ventilation may improve oxygenation and pressure palsies and mortality in patients developing early acute respiratory distress syndrome (Chalela, 2001; Guerin, 2014). Low tidal volume (<10 ml/kg BW) can benefit GBS patients due to its low probability in triggering atelectasis, partial lung collapse, and ventilator-associated pneumonia (VAP) (Ali et al., 2006). Mathematical models have been used to analyze tidal volume, tidal pressure, or tidal power to set individualized ventilation modes and to optimize targeting schemes (Van Der Staay and Chatburn, 2018).

Invasive ventilation reliant on intubation or tracheostomy is commonly utilized to solve hypoxemic or hypercapnic respiratory failure (Esteban et al., 2000). Noninvasive ventilation is less meaningful in bed-bound patients with long-term muscle weakness; even worse, it might increase the risk of emergency intubation and aggravate dysautonomia (Rabinstein, 2016). Of note is that patients with GBS might be sensitive to MV-associated hypotension due to the labile blood pressure induced by dysautonomia (Orlikowski et al., 2004). Taken together, MV is a crucial decision during the development of GBS; however, the proper time for tracheostomy, the adequate dose for antibiotic utilization, and the prediction of prolonged MV remain to be validated.

If a prolonged MV (>3 weeks) is predicted in the general ICU, tracheostomy needs to be considered immediately (Walgaard et al., 2017). Early tracheostomy potentially benefits GBS patients in several aspects: more comfort, earlier enteral nutrition, adequate oral hygiene, easier oral communication, and out-of-bed mobilization (Wang et al., 2011). Moreover, delayed tracheostomy for over 2 weeks might increase the risk of VAP, injury of laryngeal nerve/laryngeal mucosa/vocal cords, and fistula formation (Ali et al., 2006; Durbin, 2010). Nonetheless, tracheostomy may be in the early phase complicated by perioperative bleeding, esophageal perforation, and pneumothorax, and in the later phase by infection, tracheomalacia, tracheal stenosis, trachea-innominate artery fistula, and scar formation (Wijdicks et al., 2001; Mccredie and Adhikari, 2015). Multidisciplinary teamwork may make up for the abovementioned complications. For instance, anesthetists and otolaryngologists may provide technical support to reduce para-tracheostomy bleeding and post-tracheostomy tracheal stenosis. Furthermore, percutaneous dilatational tracheostomy combined with ultrasound or bronchoscopy can lower the risk of bleeding, infection, and post-tracheostomy tracheal stenosis (Esperanza et al., 2019). Prophylaxis with antibiotics may further help avert devastating complications including bacterial colonization. However, clinical monitoring lasting about 2 weeks following admission is recommended before a decision on tracheostomy is made, so as to avoid unnecessary tracheostomy and aspirations (Hughes et al., 2005). Collectively, we recommend early tracheostomy when despite the use of IVIg or PE rapid recovery is not seen, in particular for those with dysphagia, AMAN, and AMSAN.

## Supportive Care and Treatment in THE Intensive Care Unit

GBS patients need to be admitted to the ICU when one or more of these criteria are met: (a) rapid progression of respiratory muscle weaknes; (b) evolving respiratory distres; (c) severe dysautonomia or dysphagia; (d) Erasmus GBS respiratory insufficiency score (EGRIS) > 4 (Leonhard et al., 2019). Complications such as decubitus ulcers may prolong ICU stay and worsen the prognosis, which could be prevented *via* frequent repositioning (Wang et al., 2016). DVT also merits exclusive caution to prevent pulmonary embolism and sudden death (Khan, 2004). Hence, patients with severe GBS are supposed to be treated in the ICU, where adequate resources for cardia and respiratory monitoring are available (Yuki and Hartung, 2012).

### Systematic Management

Since the dysautonomia-associated cardiac arrest and bulbar palsy-triggered aspiration pneumonia are life-threatening complications, meticulous supportive care is of exclusive importance even for GBS patients with mild limb weakness. About one-fifth of GBS patients developed cardiac issues including arrhythmia and extreme hypertension or hypotension; there is also a risk for bradycardia which may cause asystole (Hughes et al., 2005). Measurement of heart rate, rhythm, blood pressure, oxygen saturation, vital capacity, blood gas and biochemistry, and swallowing should be conducted every 2–4 h if necessary, for example, when patients show progressive signs (Hughes et al., 2005). Importantly, hemodynamic and respiratory monitoring is required in monitoring patients with progressive GBS. Blood pressure monitoring could reflect the variable hemodynamics during MV and prevent cardiovascular events (Michard, 2005). Electrolyte disturbance including hyponatremia, hypernatremia, hypokalemia, hyperkalemia, and hypocalcemia should be corrected timely to avoid related cardiovascular and gastrointestinal complications (Netto et al., 2017).

In addition to ventilators, temporary cardiac pacemakers are needed for patients with severe dysautonomia or arrhythmia or extreme hypertension or hypotension (Hughes et al., 2005). Pulmonary embolism could be restricted by attenuated DVT risk, possibly *via* prophylaxis of anticoagulant medication (subcutaneous heparin), physiotherapy (positioning, respiratory therapy, and passive movements), wearing compression stockings, practicing progressive mobilization protocols, and training caregivers (Khan, 2004). Of note are medications utilized in tracheal intubation (barbiturates, benzodiazepines, narcotics, etc.) which can exaggerate dysautonomia-associated symptoms like hypotensive responses, further alerting caregivers to hypotension in GBS, especially for those who are intubated (Chalela, 2001).

### Symptomatic Management

Approximately 36% of GBS patients complain of pain 2 weeks before weakness, with 66% in the acute phase and 38% a year after disease onset (Ruts et al., 2010). Pain management in the ICU is an assessment-driven protocol-based stepwise approach. Routine pain management including pharmacological intervention (opioids, gabapentin, carbamazepine, and NSAIDs) or physical intervention (massage, music, relaxation, etc.) should be reckoned before using a sedative agent (Ruts et al., 2007; Devlin et al., 2018). Interestingly, intraepidermal nerve fiber density (IENFD), which represents unmyelinated skin nerve density, is significantly decreased in GBS patients complaining of neuropathic pain in the acute phase (Ruts et al., 2012). IENFD is also correlated with poorer Hughes functional grading scale (HFGS) scores at 6 months, scores at nadir, and clinically probable dysautonomia (Ruts et al., 2012). Indeed, circulating IgG autoantibodies are identified to attack nonmyelinating Schwann cells in 24% (56/233) GBS patients, suggesting part of the immunoreactivity in GBS is not directed against myelin but nonmyelin epitopes probably involved in Schwann cell–axon interaction (Kwa et al., 2003).

Fatigue is also a frequent complaint of GBS patients (around 40%), especially for females (74%) and those older than 50 years (Garssen et al., 2006). Physiotherapists could help mitigate patients’ fatigue and promote recovery *via* a program of strengthening, aerobic, and functional exercise (Graham et al., 2007). Other pharmacological therapies (amantadine, modafinil, etc.), cognitive behavior therapies, and psychological support could facilitate the alleviation of fatigue to some extent (De Vries et al., 2010). Noticeably, GBS-associated mental status alterations including vivid dreams, hallucinations, or psychosis-unlike ICU delirium also require attention and timely management (Cochen et al., 2005).

### Glucose Control

Glucose monitoring needs to be incorporated into the ICU supportive care to predict GBS prognosis. Higher levels of fasting plasma glucose are frequently observed in patients with cranial nerve involvement, autonomic deficit, dyspnea, and ventilator dependence, which are associated with a poorer short-term prognosis at discharge (Wang et al., 2015a). Moreover, dysglycemia was closely correlated with a neurologic disability at ICU discharge and may delay motor recovery in MV-dependent patients (Polito et al., 2019). A tight glycemic control results in reduced morbidity and mortality of surgical, medical, and pediatric ICU patients (Van Den Berghe et al., 2001; Van Den Berghe et al., 2006; Vlasselaers et al., 2009). Intensive insulin therapy is effective in lowering the incidence of ICU-acquired weakness (ICUAW) and can shorten the duration of MV for GBS patients admitted in the ICU for at least 1 week, albeit conferring a high risk of fatal hypoglycemia (Hermans et al., 2008; Zink et al., 2009).

### Unusual Conditions that Merit Extraordinary Supportive Care

Gastric–small intestine adynamic ileus was exemplified in 15% severe GBS patients, which may be triggered by immune dysfunction, dysautonomia, and immobilization (Burns et al., 2001). Routine abdominal examination including auscultation, measurement of abdominal girth, and abdominal radiography could be performed for patients with dysautonomia, those with MV reliance, and those receiving large doses of opioids (Burns et al., 2001). In this case, itopride might be beneficial. Constipation and urinary retention are foreseeable in GBS patients confined to the bed and prevented *via* the use of laxatives or bladder catheterization (Khan et al., 2011). Additionally, the expansion of setting-adapted training will be important to improve the ICU performance of ventilated patients under treatment. Psychiatrists are sometimes consulted to solve psychiatric symptoms like anxiety, stress, depression, and visual hallucination (Hillyar and Nibber, 2020).

Prolonged ventilation is associated with poor prognosis of GBS, yet patients requiring prolonged ventilation may show slow but persistent recovery for years before reaching the ability to walk independently (Van Den Berg et al., 2018). Therefore, early rehabilitation intervention ensures the medical stability and prophylactic measures to minimize long-term complications (Khan, 2004). Rehabilitation can be initiated since the acute phase with gentle strengthening involving isometric, isotonic, isokinetic, and manual resistive and progressive resistive exercises to avoid muscle shortening and joint contractures (Hughes et al., 2005). In the acute phase, patients requiring MV are generally more disabled with an extended period of disease nadir and particularly need in-patient rehabilitation (Khan and Amatya, 2012). The prolonged immobilization of ICU-admitted patients may lead to decreased blood volume and postural hypotension. Patients may need a tilt table in this case (Meythaler and Devivo, 1997). Proper bed positioning and postural changes are required for patients with weight loss and sensory loss to avoid peripheral nerve compression and decubitus ulcers (Meythaler and Devivo, 1997). High-intensity multidisciplinary ambulatory rehabilitation up to 12 months can effectively reduce disability of GBS patients at the later stages of recovery and improve the quality of life (Khan and Amatya, 2012). It is noteworthy that GBS patients after discharge may be left with psychiatric sequelae including stress, anxiety, depression, fatigue, sleep abnormalities, and visual hallucinations, which may need a multidisciplinary team approach to facilitate both physical and psychiatric recovery (Hillyar and Nibber, 2020). Although early rehabilitation is necessary for preventing ICUAW and facilitating the recovery of axonal GBS, it remains unclear, however, whether exercise-based rehabilitation, neurotrophic therapies, or acupuncture in general wards and after hospital discharge is beneficial (Lee et al., 2015; Shang et al., 2020a; Fan et al., 2020).

## Intensive Care Unit–Related Complications and Their Managements

Complications in the process of MV and ICU stay are essential parameters to predict the prognosis of GBS patients. Prolonged ICU stay (>3 weeks) may breed complex complications and increase mortality (Dhar et al., 2008). Nosocomial complications including ICUAW, hospital-acquired pneumonia (HAP), VAP, hyponatremia were considerable factors in causing death, prolonged MV (>21 days), low HFGS scores (≤3), and long hospitalization (>36 days) (Netto et al., 2017).

### Intensive Care Unit–Acquired Weakness

As the most common neuromuscular impairment which affects the clinical courses and outcomes of ICU patients (Latronico and Bolton, 2011), ICUAW mainly includes diaphragmatic weakness, critical illness polyneuropathy (CIP), and critical illness myopathy (CIM). Shrinking pressure-generating capacity and decreased diaphragmatic thickness after MV initiation synergistically trigger diaphragmatic weakness (Dres et al., 2017). Primary axonal degeneration, nerve atrophy, and compression neuropathies of peripheral sensory and motor nerves had been documented in CIP, leading to innervation and functional abnormality of the entire neuromuscular junction (Latronico et al., 1996). Numerous molecular and cellular processes including inflammation, autophagy, protein synthesis and degradation, membrane excitability, and myofibrillar interaction were involved in the pathogenesis of CIM (Sandri, 2008; Batt et al., 2013). Given that the clinical and electrophysiological features of CIP and GBS largely resemble each other (Zhou et al., 2014), the differentiation between them is complicated. Therefore, we compare the pathology, the risk factors, the clinical features, the diagnostics, and the outcomes of patients with AIDP, AMAN, and CIP in Table 3.

**TABLE 3:** Comparisons between major GBS subtypes and CIP (Zhou et al., 2014).

	AIDP	AMAN	CIP
Pathology	Demyelination of peripheral nerves with inflammation	Motor with/without sensory axonal degeneration	Axonal degeneration, nerve atrophy, and compression neuropathies of peripheral sensory and motor nerves without inflammation
Prodromal risk factors	Respiratory infection, vaccination, and monoclonal antibody	Gastrointestinal infection (mainly C. jejuni), vaccination, and ganglioside administration	Sepsis, multiple organ failure, and other critically ill conditions
Clinical features	Progressive para-/tetraparesis, sensory deficits, hypo- or areflexia, cranial nerve palsy, and progressive course	Mainly motor deficit, rarely involving cranial nerves (<20%), without pain or sensory loss, with absent tendon reflex^a^	Usually after ICU admission, fairly symmetric muscle weakness sparing cranial nerves, and less sensory deficits
Electrophysiology	Slower sensorimotor nerve conductions or CBs; excessive temporal dispersions of CMAPs; a prolonged DML or F**-**wave latency	Usually no evidence of AIDP (may show segmental conduction blocks in an early phase); show RCFs or decreased CMAP amplitudes	Normal conduction velocity and decreased amplitudes of CMAPs and SNAPs
MRI	Usually normal, occasional enhancement of peripheral nerve roots		None
CSF and serum	Elevation of CSF proteins	Elevation of CSF proteins and serum GM1, GD1a, and GM1b antibodies	None
Treatment	PE, IVIg, MV, or tracheostomy if necessary	Usually antiseptic treatment
Outcome	Progressive course with a plateau of 2–4 weeks; recovery lasting several months	Variable disease course; rapid or slow recovery	Half patients fully recover; the recovery course is variable

Abbreviations: AIDP, acute inflammatory demyelinating polyneuropathy; AMAN, acute motor axonal neuropathy; CB, conduction block; CIP, critical illness polyneuropathy; CMAP, compound muscle action potential; CSF, cerebrospinal fluid; DML, distal motor latency; GBS, Guillain–Barré syndrome; ICU, intensive care unit; RCF, reversible conduction failure; SNAP, sensory nerve action potential.

^a^ Normal or even exaggerated reflexes may exist in the early phase and atypical GBS.

Sepsis (Fan et al., 2014), hyperglycemia (Vanhorebeek et al., 2020), prolonged MV, and ICU stay increase the morbidity of ICUAW and lead to high long-term mortality (Kelmenson et al., 2017; Latronico et al., 2017). Measurement of creatine kinase (CK) (Shepherd et al., 2017) and lactate dehydrogenase (LDH) (Marshall et al., 1995) as well as their isoenzymes may be useful for monitoring the initiation and progression of CIP/CIM. ICUAW may, to some extent, mimic TRFs because of the comparable clinical features and complicate patients’ prognosis. No effective treatments for CIP/CIM are currently available; therapies including nutritional interventions, antioxidant therapy, testosterone and growth hormone therapy, and immunoglobulin are potentially beneficial to CIP/CIM (Hermans et al., 2008). Pragmatic approaches to prevent ICUAW or impede progression include aggressive treatment of sepsis, control of blood glucose, early mobilization, shortening MV-duration, and postponing parenteral nutrition during the first week of critical illness (Hermans and Van Den Berghe, 2015; Vanhorebeek et al., 2020).

### HAP and VAP

Pulmonary complications including HAP and VAP are not uncommon in critically ill patients with GBS. HAP is defined as nosocomial pneumonia developed in patients without ventilator assistance 48 h prior to infection, whereas VAP mostly arises at least 48 h after endotracheal intubation (Esperatti et al., 2010). Noticeably, patients who develop barotrauma secondary to MV also possibly end up with a prolonged ICU stay and VAP (Diaz and Heller, 2020). The bacteriology for VAP/HAP is similar, regardless of whether pneumonia is acquired during ventilation according to the type of isolates (Esperatti et al., 2010). Patients receiving intravenous antibiotics within the previous 90 days are more vulnerable to VAP/HAP induced by *Pseudomonas* or methicillin-resistant *Staphylococcus aureus* (MRSA) (Erb et al., 2016). Besides, the risk of multidrug-resistant (MDR) organism infections in VAP could be predicted by intravenous antibiotic use within 90 days, acute respiratory distress preceding VAP, septic shock or acute renal replacement at VAP onset, and >5 days of hospitalization before VAP occurrence (Erb et al., 2016). Noninvasive sampling with semiquantitative cultures of respiratory secretions and blood is recommended to make a microbiologic diagnosis in patients with VAP/HAP (Kalil et al., 2016).

Antibiotic prophylaxis could be considered for preventing nosocomial respiratory tract infections for mechanically ventilated patients in the ICU (Liberati et al., 2009). Airway clearance strategies including assisted cough or air stacking and appropriate antibiotic therapy attenuate morbidity of HAP/VAP in GBS cohorts (Koenig and Truwit, 2006; Chatwin et al., 2018). Once HAP/VAP occurs, decision-making on the use of antibiotics should be based on the duration of intubation and hospitalization, prior or current antibiotic therapy, the disease severity, and knowledge of local pathogen susceptibility patterns combined with clinical and radiographic findings (Martin-Loeches et al., 2018; Niederman, 2018). Empirical therapies for VAP include coverage of *Staphylococcus aureus* (*S. aureus*), *Pseudomonas aeruginosa*, and other Gram-negative bacilli, while antibiotics against *S. aureus* are recommended in the HAP empiric regimen (Kalil et al., 2016). Notably, antibiotic therapy in a fixed course (7–8 days) may reduce the emergence of MDR organisms without increasing the risk of adverse clinical outcomes for patients with VAP, better than a prolonged course (10–15 days) (Pugh et al., 2015). The key clinical features of VAP and HAP are displayed in Table 4.

**TABLE 4:** Differences and Similarities between HAP and VAP.

	HAP	VAP
Definition	Nosocomial pneumonia developed in patients without ventilator assistance 48 h prior to infection (Zaragoza et al., 2020)	Pneumonia arises at least 48 h after the endotracheal intubation (Pahal et al., 2020); patients with ventilator-associated events including barotrauma or VALI may also end up with VAP (Diaz and Heller, 2020)
Pathogen	Early phase: Gram-negative bacilli, MRSA, *Streptococcus pneumoniae*, *Mycoplasma pneumoniae*, *Chalamydophila pneumoniae*, *Haemophilus influenzae*, *Haemophilus parainfluenzae*, etc. Later phase: MRSA, MDR organisms, *Acinetobacter*, *Pseudomonas aeruginosa*, etc.	Similar to HAP, more likely to be triggered by nonfermenting and enteric Gram-negative bacilli, MRSA
Clinical symptoms	Fever (>38°C) or hypothermia (<36°C), purulent sputum, decreased blood oxygen saturation, rales or bronchial breath sounds, increased oxygen/ventilator requirement	Fever (>38°C) or hypothermia (<36°C), increased respiratory secretion, new-onset tachypnea/dyspnea, rales or bronchial breath sounds, increased minute ventilation, bradycardia, etc.
Laboratory workups	Leukocytosis (>12,000) or leukopenia (<4,000), or positive results from sputum/blood cultures	Similar to HAP
Radiology	New or progressive infiltrates, cavitation, or consolidation	Similar to HAP
Diagnosis	Based on clinical symptoms, signs, and history, X-ray or CT, bronchoscopy or sputum/blood culture results	Similar to HAP
Treatment	Empirical use of antibiotics based on local pathogen susceptibility patterns, clinical symptoms/signs, and radiographic findings before antibiotic susceptibility results are available	Similar to HAP
Prevention	PPE and prophylactic antibiotics for patients with high risks	Avoid prolonged MV duration, early tracheostomy if necessary, and prophylactic antibiotics if necessary

Abbreviations: CT, computed tomography; HAP, hospital-acquired pneumonia; MDR, multidrug-resistant; MRSA, methicillin-resistant *Staphylococcus aureus*; MV, mechanical ventilation; PPE, personal protective equipment; VALI, ventilator-associated lung injury; VAP, ventilator-associated pneumonia.

### Rare Complications in Guillain–Barré Syndrome

Patients with severe GBS are particularly susceptible to pressure palsies for two reasons: (a) loss of functional position in GBS patients leads to an imbalance between flexor and extensor muscles and (b) inflamed nerves are susceptible to pressure injury (Chalela, 2001). Entrapment neuropathy is not uncommon in the ICU (Kalb, 2014). Isolated nerve entrapment may present as focal pain or weakness. Prevention *via* adequate awareness and proper limb positioning is of utmost importance for entrapment neuropathy.

ICU stay or dysautonomia may also trigger CNS complications in the form of posterior reversible encephalopathy syndrome (PRES), which has been occasionally reported in adult and adolescent patients (Van Diest et al., 2007; Rigamonti et al., 2012; Chen et al., 2015; Nabi et al., 2016). Patients with PRES do not require pharmacological interventions, whereas some may need symptomatic therapies to solve PRES-associated epilepsy or aggressive elevated blood pressure (Hobson et al., 2012). ICU delirium may also occur after a long ICU stay, which is usually managed *via* pharmacological methods (haloperidol, olanzapine, etc.) and nonpharmacological methods (multicomponent strategy, geriatrics consultation, reduced benzodiazepine use, etc.) (Girard et al., 2008). Besides, 48% of patients had syndrome of inappropriate antidiuretic hormone secretion (SIADH) during the GBS course, and those individuals showed lower Medical Research Council (MRC) scores, longer hospital stay, higher risks for MV dependence, and more likeliness to require PE (Saifudheen et al., 2011). Symptomatic management (i.e., water restriction and hypotonic saline) or vaptan prescription might benefit these patients (Zietse et al., 2009).

## Concluding Remarks and Future Perspectives

Managements of severe GBS in the ICU setting are usually multidisciplinary and even more complex in continuously evolving conditions. The intensive care and treatment is better provided by neurointensivists so as to precisely monitor the progression of GBS. Currently, PE and IVIg remain the mainstay of GBS treatment. Despite research displaying the superiority of IVIg to PE in MV-dependent GBS patients (Charra et al., 2014), the evidence quality is very low. No evidence supports the combinational use of IVIg and PE for severe GBS patients as well. As such, neurointensivists still need to weigh the cost-effectiveness ratio and the reward-to-risk ratio of IVIg or PE or a combination of the two, especially in the ICU setting. Future large sample size RCTs are needed to address treatment dilemmas like mild cases, variant forms of GBS (i.e., MFS), TRF, when the onset of weakness was more than 2 weeks ago, or when patients do not improve or even progress after initial treatment. Efficacy of small-volume PE, double filtration plasmapheresis, a second dose of IVIg, and very early use of steroids merits further investigation. Severe GBS patients have high risks of respiratory failure due to respiratory muscle weakness, dysphagia and dysautonomia, and nosocomial infections. Intubation and invasive MV effectively relieve respiratory compromise which, when indicated, should be initiated as early as possible so as to avoid emergency intubation. Early tracheostomy in severe patients, especially when improvement is trivial after immunotherapy, should be considered, which may potentially benefit patients in terms of comfort and communication. Complications of GBS patients in the ICU setting, that is, DVT, ICUAW, HAP/VAP, PRES, ICU delirium, and SIADH should be diagnosed timely and treated accordingly, although emphasis lies in prophylaxis. A multidisciplinary team is needed to assist intensive care for severe GBS cases to avert life-threatening complications. Moreover, rehabilitation and psychological support are also emphasized in both ICU-recruited and discharged patients.
